# Compassion As an Intervention to Attune to Universal Suffering of Self and Others in Conflicts: A Translational Framework

**DOI:** 10.3389/fpsyg.2020.603385

**Published:** 2021-01-11

**Authors:** S. Shaun Ho, Yoshio Nakamura, James E. Swain

**Affiliations:** ^1^Department of Psychiatry and Behavioral Health, Stony Brook University, Stony Brook, NY, United States; ^2^Department of Anesthesiology, Division of Pain Medicine, Pain Research Center, University of Utah School of Medicine, Salt Lake City, UT, United States

**Keywords:** meditation, neuroimaging, Bayesian, free energy principle, Buddhism, compassion (*karuna*), conflicts, *lojong*

## Abstract

As interpersonal, racial, social, and international conflicts intensify in the world, it is important to safeguard the mental health of individuals affected by them. According to a Buddhist notion “if you want others to be happy, practice compassion; if you want to be happy, practice compassion,” compassion practice is an intervention to cultivate conflict-proof well-being. Here, compassion practice refers to a form of concentrated meditation wherein a practitioner attunes to friend, enemy, and someone in between, thinking, “I’m going to help them (equally).” The compassion meditation is based on Buddhist philosophy that mental suffering is rooted in conceptual thoughts that give rise to generic mental images of self and others and subsequent biases to preserve one’s egoism, blocking the ultimate nature of mind. To contextualize compassion meditation scientifically, we adopted a Bayesian active inference framework to incorporate relevant Buddhist concepts, including mind (buddhi), compassion (*karuna*), aggregates (*skandhas*), suffering (*duhkha*), reification (*samaropa*), conceptual thoughts (*vikalpa*), and superimposition (*prapañca*). In this framework, a person is considered a Bayesian Engine that actively constructs phenomena based on the aggregates of forms, sensations, discriminations, actions, and consciousness. When the person embodies rigid beliefs about self and others’ identities (*identity-grasping beliefs*) and the resulting *ego-preserving bias*, the person’s Bayesian Engine malfunctions, failing to use prediction errors to update prior beliefs. To counter this problem, after recognizing the causes of sufferings, a practitioner of the compassion meditation aims to attune to all others equally, friends and enemies alike, suspend identity-based conceptual thoughts, and eventually let go of any identity-grasping belief and ego-preserving bias that obscure reality. We present a brain model for the Bayesian Engine of three components: (a) Relation-Modeling, (b) Reality-Checking, and (c) Conflict-Alarming, which are subserved by (a) the Default-Mode Network (DMN), (b) Frontoparietal Network (FPN) and Ventral Attention Network (VAN), and (c) Salience Network (SN), respectively. Upon perceiving conflicts, the strengthening or weakening of ego-preserving bias will critically depend on whether the SN up-regulates the DMN or FPN/VAN, respectively. We propose that compassion meditation can strengthen brain regions that are conducive for suspending prior beliefs and enhancing the attunements to the counterparts in conflicts.

## Introduction

Conflicts are inevitable in a society whose members have diverse interests and ideologies. Unfortunately, political polarization and inter-group animosity has increased in the United States in the past decade (Pew Research Center, 2014). Even when facing the pandemic of COVID-19, which is blind to one’s viewpoint, the partisan gap has grown even wider on nearly all important matters (Pew Research Center, 2020a,b). Due to built-in self-organizing algorithms, social media users tend to encounter messages that are similar to what they already liked or viewed. Since the cognitive costs for *attuning to counterparts* in conflicts are much higher than those for sticking to one’s own ideas, the likelihood of attuning to people with different views is diminishing.

As social conflicts increase in frequency and intensity, their adverse effects on personal well-being also increase in extent and severity. Conflict-related adversities in early life are associated with poor health and reduced well-being. For example, complete social isolations since birth caused great harms (Harlow et al., 1965); adverse childhood experiences (ACEs) predicted adulthood psychopathology (Anda et al., 2006), incoherent social relationships (Tyrka et al., 2009), suicidality (Dube et al., 2001), and other mortality risk factors (Felitti et al., 1998); poor quality in social relationships have detrimental effects on health (Umberson and Montez, 2010) and positive association with cellular aging (Uchino et al., 2012); an animal model suggested that when conflicts resulted in social defeat, individuals are prone for substance abuse and social emotional disorders (Hammels et al., 2015). Across mental disorders that conflict-induced adversities may aggravate, the failure of updating beliefs is very common (Kube and Rozenkrantz, 2020). Thus, rigid beliefs may be a manifestation or cause of post-conflict malfunctioning.

Notably, not all individuals subjected to defeats in social conflicts are devastated. When people with childhood adversity became adults, while about two-thirds of them suffered from various symptoms of substance use, psychopathology, impaired functioning in professional social realms, one-third of them were resilient, doing even better than low-adversity controls by showing more personal competence, determination, strong social support from a domestic partner, and reliance on faith and prayer (Werner, 1992). Likewise, many studies have documented that spirituality and intimate social support played crucial roles in the post-adversity bifurcation between resilient and worsened well-being (Feiring et al., 1996; Leskela et al., 2002; Wilson et al., 2006; Sippel and Marshall, 2011; Saraiya and Lopez-Castro, 2016; Lahav et al., 2017). Mother–child attunement is considered a foundation of resilience (Feldman, 2020).

We postulate that one of the key ingredients in the post-conflict resilience afforded by spirituality and positive social relationship is attunement, which can be construed from two perspectives: (1) In psychotherapy, attunement refers to a kinesthetic and emotional sensing of others knowing (tuning-in) their rhythm, affect and experience. This is a two-part process of communion of interpersonal contact—beginning with *empathy*, i.e., being sensitive to and identifying with the other person’s sensations, needs or feelings, and then communication of that sensitivity to the other person (Erskine, 1998). (2) In Buddhism, as it will become clear below, attunement refers to both the function of mind and meditative trainings of mind, as Arya Shantideva said in *A Guide to the Bodhisattva*’*s Way of Life* (hereafter, GBWL):

*“Thus, because he loves to pacify the pains of others, he whose mind is attuned in this way, would enter even the deepest hell, just as a wild goose plunges into a lotus pond.”* (Shantideva, c. 700/1979)

An authentic instruction on this particular verse in GBWL is that “if you keep on doing it, your mind will be trained in that way. It will become part of your habit, your way of life. You function that way; your mind is attuned. If you do so, it will be like birds that are attracted to a beautiful lotus lake. No matter how far they have to fly, all the birds and animals will try to come to the lotus lake… What does that mean? When you can develop compassion, caring for people, they will be with you. They will help you and protect you, because you serve them.” (Gelek Rimpoche, 2008). Notably, these two perspectives on attunement are highly coherent; after all, it is the mind that makes the attunement in psychotherapy possible.

Would empathy and compassion help us deal with conflicts? Induced perspective-taking can facilitate autonomic attunement and reduced negative affect after conflicts (Nelson et al., 2017). In a systematic review on the roles of affective empathy, personal distress, and compassion in interpersonal and intergroup conflicts, it was found that empathy, compassion, and training thereof may facilitate the resolution of interpersonal and intergroup conflicts and overcome otherwise limiting factors such as intergroup empathy bias, identifiable victim effect, and reduced motivation for empathy toward out-group members (Klimecki, 2019).

Can compassion be cultivated through non-religious training to safeguard post-conflict well-being? As well-being can be promoted by mental skills related to inner experiences, there has been an emerging interest on cultivating compassion toward self and others (Gilbert, 2019). For examples, studies of loving-kindness meditation demonstrated that a brief practice was able to increase feelings of social connectedness and affiliation toward strangers (Hutcherson et al., 2008) and that repeated weekly training sessions led to increase positive emotions, mindfulness, feelings of purpose in life and social support as well as to decrease illness symptoms (Fredrickson et al., 2008).

While a comprehensive review of compassion training is beyond the scope of this paper, the literature suggested that compassion-based interventions produced moderate effect sizes for reduced suffering and improved life satisfaction, suggesting the potential benefits of compassion-based interventions on a range of outcomes related to well-being (Kirby, 2017; Kirby et al., 2017). Future research is required to study the effects of Buddhism-derived compassion trainings on conflict resolution.

While perhaps nobody would dislike being a recipient of attunement from others, i.e., being heard and understood, attuning to others, especially to counterparts who stand in conflicts with oneself, may be resisted or even dismissed under some circumstances. Such resistance to attune to unfriendly others may result from fears of being overwhelmed by others’ suffering, diversion of one’s own limited resources, being exploited, losing the competition, or irrationality that may compromise rule-of-law in society; see Mascaro and Gilbert (2017) for a model of fears, blocks, and resistances of compassion. All these fears point to an attack against one key ingredient of empathy, i.e., identifying with others’ pains as if they are your own (Bloom, 2017).

If attunement to others in suffering already has drawbacks, why should anyone attune to counterparts who stand against him or her? Buddhist philosophy provides an answer to this provocative question: If one recognizes *the ultimate nature of mind*, then one can see it is one’s own *ego-preserving bias*, not the counterparts in conflicts, that blocks one’s equal attunement to self and others and harms one’s own post-conflict well-being. That is, any discontents against equal attunement to self and others will cease to exist, *if and only if* one truly recognizes the ultimate nature of mind.

Against this backdrop, we aim to introduce relevant Buddhist concepts in the present work in order to identify an entry point for attunement-oriented interventions to safeguard personal well-being despite inevitable conflicts, although such personal intervention is by no means a substitute for more socially active processes such as better journalism, better accountability of politicians, and better rule of law. We identified an entry point at the point of bifurcation between two incompatible post-conflict responses in an individual: (a) attuning to the counterparts’ perspectives and needs *despite* the conflicts and (b) blocking the attunement to the counterparts *due to* the conflicts. We hypothesize that:

Hypothesis 1: A form of bias, namely ego-preserving bias, can prevent a person from attuning to those whose perspectives conflict with his or her own.
Hypothesis 2: A form of compassion meditation designed to cultivate equal attunement to universal suffering of self and others can ultimately eliminate the ego-preserving bias.

Given that people’s normal responses to conflicts are driven by the brain that is of limited capacity and irrationalities, it has been noted that “if we are to become more than just actors in ancient archetypal dramas, it requires us to take responsibility to understand our brains” (Gilbert, 2015, p. 265). We wish to add that we also need to take responsibility to understand the ultimate nature of mind. It is in this spirit that we put together this multi-faceted paper with the following sections: Section “Introduction,” our post-conflict bifurcation hypotheses, described above; Section “Parsing Our Hypotheses in Terms of Buddhist Concepts,” Buddhist notions of the ultimate nature of mind and key processes through which ego-preserving bias can entrap the mind in universal sufferings; Section “Compassion Meditation and How It Maintains Attunement to Others,” Buddhist science and practice of compassion to elucidate the ultimate nature of the mind and to remove ego-preserving bias by attuning to friends, enemies, and strangers’ universal suffering equally; Section “Incorporating Buddhist Concepts in a Bayesian Active Inference Framework,” how the Buddhist notions of “person” can be understood in a Bayesian active inference framework that has powerfully accounted for various forms of complex adaptive systems in the literature; Section “A Brain Model for the Social Cognition, Embodied Identity-Grasping Beliefs, and Compassion Meditation Effects,” a putative brain model of the bifurcation of post-conflict responses.

## Parsing Our Hypotheses in Terms of Buddhist Concepts

Buddha Gotama gave his first teaching on the foundation of Buddhism, Four Noble Truths, namely, (1) Truth of Suffering, (2) Truth of the Origin of Suffering, (3) Truth of Cessation (of suffering), and (4) Truth of the Path (to cessation of suffering), that can be recognized by those noble persons who directly perceive ultimate reality (Tenzin Gyatso, 1997). We consider that our first hypothesis lies in the scope of the second Noble Truths, and our second hypothesis in the scope of the fourth Noble Truths.

To parse our hypotheses in terms of Buddhist concepts, we introduce Buddhist concepts mainly according to three authoritative texts, (1) the Science and Philosophy in the Indian Buddhist Classics, Volume II: the Mind (hereafter, SPIBC-M) (Tenzin Gyatso, 2020), (2) the Princeton Dictionary of Buddhism (hereafter, PDB) (Buswell and Lopez, 2013), and (3) Arya Shantideva’s GBWL (Shantideva, c. 700/1979). The transliteration of Sanskrit and Tibetan is printed in all-upper-case and all-lower-case-italic, respectively.

The backbone of our hypotheses is reflected in the following verses from Arya Shantideva’s GBWL (BODHISATTVACHARYAVATARA) (Shantideva, c. 700/1979):

Ch. 6, V.52

Since my mind is not physical, in no way can anyone destroy it. But through its being greatly attached to my body, it is caused harm by suffering.

Ch. 8, V.90

First of all, I should make an effort to meditate upon the equality between self and others: I should protect all beings as I do myself because we are all equal in (wanting) pleasure and (not wanting) pain.

Ch. 8, V.107

Thus, because he loves to pacify the pains of others, He whose mind is attuned in this way, would enter even the deepest hell, just as a wild goose plunges into a lotus pond.

Ch. 8, V.115

Through acquaintance has the thought of “I” arisen toward this impersonal body; So, in a similar way, why should it not arise toward other living beings?

The key concepts in the above verses that will be covered in the following sections include:

(1) In ultimate truth, *mind is not physical*, and therefore it is neither destroyable nor vulnerable for sufferings. We will discuss this notion in Sections “The Nature of Mind and The Ultimate Nature of All Phenomena.”
(2) Through certain *acquaintance* (and *misattribution*) processes, mind can be attached to an otherwise impersonal (selfless) collection of aggregates, giving rise of a false identity of “I” that is bound to suffer. We will discuss these processes in Sections “Buddhist Concepts of Person and Its Impersonal Processes and How VIKALPA and PRAPAÑCA Prevent Attunements to Others.”
(3) Recognizing the faults of misattribution of “I,” one who aspires the ultimate excellence will attune the mind upon the equal identification with self and others and the nature of mind to pacify distorted conceptions. We will discuss these notions in Section “Compassion Meditation and How It Maintains Attunement to Others.”

### The Nature of Mind

We consider the concept of attunement as synonymous with “awareness,” the function of mind. The notion of “attunement to a person” means awareness of a person’s mental and sense awareness manifesting in the person’s five aggregates (SKANDHAS), described later, which encompass the person’s physical and social attributes, e.g., sex, age, nationality, religion, social status, and non-physical attributes, e.g., mental awareness and mental factors, e.g., feelings, intentions, perspectives, memories etc.

The nature of mind is clear (as its ultimate nature) and aware (as its function), as explained in SPIBC-M (Tenzin Gyatso, 2020):

“*Generally, in the context of Buddhist texts, the terms cognition [or comprehension or discernment] (Sanskrit: BUDDHI, Tibetan: blo), consciousness (JNANA), and awareness (SA$\underset{\cdot }{\text{M}}$VITTI) are all treated as coextensive or synonymous. The nature of cognition is stated to be awareness, and the nature of consciousness is said to be clear (or luminous) and aware. “Clear” here expresses the essential nature of consciousness, and “aware” expresses its function. “Clear” also indicates: (1) that consciousness is beyond the nature of matter, which is characterized as tangible and obstructive, so it is clear in nature; (2) that just as reflections appear in a mirror, any internal or external object whatsoever — good or bad, pleasant or unpleasant — can appear in consciousness, so consciousness is luminous in that it illuminates objects; and (3) that the essential nature of consciousness is not contaminated by the stains of mental afflictions such as attachment, so its nature is clear or luminous”* (Tenzin Gyatso, 2020).

Buddhist teachers often likened the nature of the mind to a clear lamp shade (Geshe Rabten, 1997; Gelek Rimpoche, 2005). In this metaphor, a clear lamp shade is colorless (clear) and any object that the mind perceives is like a light bulb in the clear lamp shade, which can color the clear lamp shade with the color of its light, e.g., the lamp shade’s color becomes red when a light bulb sheds red light to the shade. However, just like the light bulb can never stain the lamp shade, the object perceived by the mind can never stain the mind. Thus, the mind (lamp shade) returns to its colorless clarity as soon as the object (light bulb) is turned off.

In Buddhism, there is a distinction between the ultimate nature of mind (CITTA) and the mental factors (CAITTA). The former is not causally related to any objects other than being aware of them; the latter are causally defined in terms of their functional relationships with other factors, e.g., intention, feeling, discrimination, thinking, etc. Although the ultimate nature of mind is irreducible to physical forms, like a clear lamp shade is irreducible to light bulbs, the mind can be entrapped in an impersonal Bayesian active inference process and the brain, as explained in Sections “Incorporating Buddhist Concepts in a Bayesian Active Inference Framework” and “A Brain Model for the Social Cognition, Embodied Identity-Grasping Beliefs, and Compassion Meditation Effects.”

### The Ultimate Nature of All Phenomena

The third Noble Truth, the cessation of suffering, refers to the realization of ultimate nature of all phenomena (Nagarjuna, 1995). The key to this realization is to understand the notion of dependent origination (PRAT$\bar{\text{I}}TYASAMUTP\bar{\text{A}}$DA), and thus it has ontological, epistemological, and soteriological implications. In Buddha Gotama’s earliest teaching, PRAT$\bar{\text{I}}TYASAMUTP\bar{\text{A}}$DA was explained as “*from the arising of this, that arises; from the cessation of this, that ceases,”* Later, Arya N$\bar{\text{a}}g\bar{\text{a}}$rjuna, the founder of the MAHAYANA MADHYAMAKA school, expounded this notion further that everything comes into existence in dependence on something else, with such dependence including (1) the dependence of an effect upon its cause, (2) the dependence of a whole upon its parts, and (3) the dependence of an object on the consciousness that designates it. The MADHYAMAKA school sees a necessary relation between dependent origination and emptiness (Ś$\bar{\text{U}}NYAT\bar{\text{A}}$), arguing that because everything is dependently arisen, everything is empty of independence and intrinsic existence (SVABH$\bar{\text{A}}$VA) (Buswell and Lopez, 2013). When a person directly perceives the union of dependent origination and emptiness, the person is considered to become totally enlightened, as a buddha (Nagarjuna, 1995; Tenzin Gyatso, 2009).

In sum, as far as the ultimate truth is concerned, the nature of mind, which is clear and aware, and the nature of all phenomena, which is dependent origination, are both empty of intrinsic existence, i.e., the ultimate nature of what is observed is not identical to what it appears in phenomena.

### Buddhist Concepts of Person and Its Impersonal Processes

The concept of person, as in the “attunement to a person,” does not refer to an indivisible entity, but a collection of five aggregates (SKANDHAS) functioning in an *impersonal* (selfless) process of dependent origination, including: (1) materiality or form (R$\bar{\text{U}}$PA), (2) sensations or feelings (VEDAN$\bar{\text{A}}$), (3) perception or discrimination (SA$\underset{\cdot }{\text{M}}$JÑ$\bar{\text{A}}$), (4) conditioning factors or volitional actions (SA$\underset{\cdot }{\text{M}}SK\bar{\text{A}}$RA), and (5) consciousness (VIJÑ$\bar{\text{A}}$NA). According to PDB, in this impersonal process of perception-action cycle, consciousness (VIJÑ$\bar{\text{A}}$NA) in one of six modalities (visual, auditory, olfactory, gustatory, bodily, and mental consciousness) occurs as the result of the interactions between an internal sense base (INDRIYA) and an external sense object ($\bar{\text{A}}$YATANA) in each modality. When the internal sense base, external sense object, and consciousness co-occur in a contact (SPARŚA), it leads to the feeling (VEDAN$\bar{\text{A}}$) of that contact as pleasant, unpleasant, or neutral. At that point, however, this *impersona*l process can be intruded by a sense of ego, which refers to the perspective of a conceited, self-affirming “I” when all sensory experiences are perceived in relation to “I” itself. After the intrusion of ego, this process becomes egoistically personal, whereby what one feels, one perceives (mediated by a prior mental image) (SA$\underset{\cdot }{\text{M}}$JÑ$\bar{\text{A}}$); what one perceives, one thinks about (VITARKA); and what one thinks about, one proliferatively conceptualizes (PRAPAÑCA), all infused with the sense of self. As a result, the person lives in a bondage to SA$\underset{\cdot }{\text{M}}S\bar{\text{A}}$RA, when everything that can be experienced in the past, present, and future is bound together into a tangled network of concepts, all tied to oneself and projected into the external world as craving (T$\underset{\cdot }{\text{R}}$$\underset{\cdot }{\text{S}}$$\underset{\cdot }{\text{N}}$$\bar{\text{A}}$), conceit (M$\bar{\text{A}}$NA), and wrong views (D$\underset{\cdot }{\text{R}}$$\underset{\cdot }{\text{S}}$$\underset{\cdot }{\text{T}}$I) (Buswell and Lopez, 2013).

The conceptual proliferation (PRAPAÑCA), as described above, refers to the tendency to superimpose the perspective of ego throughout all of one’s sensory experience via the medium of concept (Buswell and Lopez, 2013). PRAPAÑCA develops when the mind is already infused with conceptual thoughts (VIKALPA) that can be proliferated. Conceptual thoughts (VIKALPA) operate through the medium of generic images (S$\bar{\text{A}}M\bar{\text{A}}NYALAK\underset{\cdot }{\text{S}}A\underset{\cdot }{\text{N}}$A), i.e., mental images or qualities that are generic to a class of phenomena, as opposed to those specific qualities that are unique to a given object. Closely related to VIKALPA is a mental factor of thinking or applied thought (VITARKA), which refers to a mental factor of an initial engagement of or inquiry into an object that is not sufficient for a full-blown VIKALPA (Tenzin Gyatso, 2020). Both conceptual thoughts (VIKALPA) and conceptual constructions (KALPAN$\bar{\text{A}}$) are often contrasted against the direct perception (PRATYAK$\underset{\cdot }{\text{S}}$A), especially yogic direct perception (YOGIPRATYAK$\underset{\cdot }{\text{S}}$A), in which reality is perceived directly without the medium of mental images (Buswell and Lopez, 2013).

### How VIKALPA and PRAPAÑCA Prevent Attunements to Others

It is important to note that mental awareness is one of six kinds of consciousnesses, in addition to five sense consciousness, i.e., vision, audition, olfaction, gustation, and bodily senses, which are afforded by different organs in the body (Tenzin Gyatso, 2020). The percepts in sense consciousness are direct perceptions when three factors, an object (say a cup), sense organ (eyes), and a top-down model from a previous moment come in contact. As the sixth kind of consciousness, mental awareness is pervading but distinct from the sense consciousnesses, as mental awareness is non-organic (not bound to an organ) and non-local (not bound to any perceived object at a specific locus in time and space). Nonetheless, mental awareness can be conceptual, mediated by mental images, which can obscure direct perceptions. When a person’s mental awareness becomes nothing but direct perception of any phenomenon as it is, i.e., non-conceptual (unmediated by mental images), the person is a buddha (Tenzin Gyatso, 2020).

As Dharmakirti said, the nature of the mind is luminous clarity and the stains are adventitious (Tenzin Gyatso, 2020), so attunement to one’s counterpart can occur despite conflicts, because mental awareness is not bound to attributes that one’s sense and conceptual consciousnesses have designate as conflicts. That is, conceptual thoughts that obstruct direct perceptions (attunement) of self and counterparts are only adventitious “stains” to the mind. Since the primary causes of mind-entrapping “stains,” VIKALPA and PRAPAÑCA, are not inherently existing, they can be ultimately eradicated through systematic methods.

To promote the awareness of VIKALPA and PRAPAÑCA, we hereby discuss how VIKALPA and PRAPAÑCA are related to our first hypothesis. According to Arya Asanga, PRAPAÑCA is a process that can proliferate and embody eight types of conceptual thoughts (VIKALPA) that are supported by three levels of substances, i.e., vehicles of conceptual thoughts (Asanga, 2016). The eight types of VIKALPA are as follows.

Type 1. The conceptual thought that conceives of an essential nature.
Type 2. The conceptual thought that conceives of a distinguishing characteristic.
Type 3. The conceptual thought that grasps a collection (of distinguishing characteristics) as a separate entity.
Type 4. The conceptual thought that conceives of an “I.”
Type 5. The conceptual thought that conceives of entities as being “mine.”
Type 6. The conceptual thought that conceives of entities as being agreeable.
Type 7. The conceptual thought that conceives of entities as being disagreeable.
Type 8. The conceptual thought that conceives of entities as being neither agreeable nor disagreeable, thus leading to an attitude of indifference toward it.

VIKALPA are embodied in three levels of substances (vehicles), which are developed in the order described below (Asanga, 2016):

Level I: this level of substance provides a basis for the first three types of VIKALPA (Types 1–3). These three types are beliefs that a property exists deterministically in a specific form, identical to its observed appearance, independent of specific circumstances. This substance serves as the basis of PRAPAÑCA, a proliferating process that superimpose ego onto an impersonal process, through which the next levels of substances, and the VIKALPA supported by them, develop.
Level II: this level of substance is the basis of the next two types of VIKALPA (Types 4–5). The view of ego is one that erringly grasps a self that is separate from a collection of perishable events, i.e., the “I” who affirms itself to exist, which is the root of all ego-centric views. The egoistic views feed an egoistic conceit—a sense of entitlement to justify an event’s value as “good” or “bad” according to one’s own views, self-affirmingly, e.g., “It’s good or bad (because I think so).”
Level III: this level of substance is the basis of the last three types of VIKALPA (Types 6–8)—which evaluate entities as being agreeable, disagreeable, or neither and give rise to craving, hatred, or ignorance according to circumstances, respectively.

In the context of conflicts, we postulate that the eight types of VIKALPA and the three levels of substances can account for the identity-dependent social cognition, as follows:

The first three types of VIKALPA (Types 1–3) point to the fundamental reification (SAM$\bar{\text{A}}$ROPA), i.e., a mistaken attribution to an object of a quality that the object does not in fact possess (Buswell and Lopez, 2013). When dealing with complex social interactions, the subtle VIKALPA compels a person to think of characteristics that he or she believes to be identical to another person’s “true nature,” forming a mis-belief that the true nature of a person is identical to certain attributes based on *generic images of a person*, such as stereotypes of gender, race, religion, nationality, etc.

The middle two types of VIKALPA (Types 4 and 5) point to a further reduction of a person to divide up the world based on available existing identities, i.e., the identity of the observing subject “I” that is different from the identities of the observed objects, including other people. Based on the level of similarity between the identities of self and others, other people whose generic images are relatively similar to “I” are reduced to the identity of “my people” (in-group), and those others whose generic images are distinct from “I” are reduced to the identity of “not my people” (out-group). These identities of “I” and “my people” are *over-reductions* because nuanced information that is not represented by generic images, such as feelings, thoughts, and relationships, is disregarded erroneously in the construction of the VIKALPA. We refer to these types of VIKALPA as identity-grasping beliefs. As beliefs, these embodied identity-grasping thoughts are always biased to serve the ego, *self-righteously*, disregarding any counter evidence suggesting that this may be a misbelief (wrong view).

The last three types of VIKALPA point to the generic images of friends, enemies, and neutral persons who can serve as objects that can invoke feelings of attraction, repulsion, and indifference, and these feelings lead to thoughts and actions that behaviorally condition a person in a network of concepts. These types of VIKALPA are cognitive bases of ego-preserving bias. A person with ego-preserving bias will strive to maintain his or her prior beliefs related to self and others, denying or neglecting any data that suggest nuanced information inconsistent with his or her own identity-grasping beliefs.

Thus, as our first hypothesis, we hypothesize that ego-preserving bias can prevent a person from attuning to those whose perspectives conflict with his or her own. If someone firmly holds an identity-grasping belief, any conflicts between self and others can be over-reduced to an inevitable battle between good and evil categorically (however fictitious that may be), and this person will self-affirm his or her own view and act to preserve his or her own egocentric interest. In such ego-preserving bias, there is no space or need to attune to the counterparts’ expectations and interests or their fleeting thoughts and feelings.

## Compassion Meditation and How It Maintains Attunement to Others

“If you want others to be happy, practice compassion; if you want to be happy, practice compassion.” by Khunu Lama Tenzin Gyaltsen, according to HHDL Tenzin Gyatso

In Buddhism, compassion (KARUNA), i.e., the wish that others be free from suffering, and benevolence (MAITRI), i.e., the wish that others be happy, are like two sides of the same coin (Buswell and Lopez, 2013). In this section, we will introduce Buddhist compassion meditation and explain how it can subdue ego-preserving bias, hence making it possible to attune to self and others equally and make self and others happy (being well).

### Three Types of Suffering to Be Recognized in Compassion Meditation

As the wish for others to be free from suffering, the meaning of compassion is incomplete in the absence of the recognition of suffering. According to Buddhism (Tenzin Gyatso, 2020), when a person’s mental awareness is obscured by conceptual thoughts (VIKALPA), or conceptual construction (KALPAN$\bar{\text{A}}$), life is nothing but SA$\underset{\cdot }{\text{M}}S\bar{\text{A}}$RA—a bondage to three types of suffering (DUHKHAS), as follows (Buswell and Lopez, 2013):

(1) Misery caused by physical or mental suffering (DUHKHADUHKATA).
(2) Misery caused by change (VIPARINAMADUHKHATA), i.e., pleasant sensations may be a cause of suffering because they do not persist and eventually turn into pain.
(3) Misery caused by conditioning (SAMSKARADUHKHATA), i.e., sensations that are neither painful nor pleasant may still be a cause of suffering because they are impermanent and thus undependable; because of past actions (KARMAN), suffering may always occur unexpectedly in the next moment.

The three types of suffering can be named differently in social domains (Buswell and Lopez, 2013):

(1) The misery caused by mental suffering can manifest as “the suffering of being associated with persons and things one dislikes,” which involves VIKAPLA of thinking a generic image of enemies.
(2) The misery caused by change can manifest as “the suffering of being separated from persons and things one likes,” which involves VIKALPA of thinking a generic image of friends.
(3) The misery of conditioning can be summarized as “the suffering of not getting what one wants,” which involves the dissatisfaction despite the push-and-pull between the VIKALPA of thinking enemies, friends, and anyone in between.

We point to the following ingenious animal study as a model to illustrate the three types of suffering. In this study, the rats were implanted with electric stimulation in the lateral hypothalamus and they were placed in a shuttle box, with a lever on either end of the box. Pressing the lever in one end could turn on electric stimulation and the lever in the other end could turn it off. Electric stimulation of the lateral hypothalamus is known to evoke appetite and other motivational behaviors. Surprisingly, after the rats learned to press the levers to turn the stimulation on or off, the rats ran between two ends constantly, alternating between turning on the stimulation on one end and turning it off on the other end (Mendelson and Freed, 1973). Thus, neither of the presence (“on”) and absence (“off”) of electric stimulation in the lateral hypothalamus can fully satisfy the rats such that they can stop running.

Using this animal study as a metaphor, a person entrapped in VIKALPA and PRAPAÑCA is like the rats in the study described above. Say, evoking a mental image of “friend” or “enemy” is like turning on the electric stimulation in the rats, and the cessation of this mental image is like turning off the stimulation in the rat. Whether turning on the stimulation is appetitive (like thinking of a mental image of “friend”) or aversive (like thinking of a mental image of enemy), the stimulation or cessation of these mental images are miseries caused by suffering or miseries caused by change (the first two types of suffering); and the conditioning that causes the alternation between turning on and off a mental image is misery caused by conditioning actions (the third type of suffering).

### Buddhist Compassion Meditation - *Lojong*

*Lojong* (or *blo sbyong*) is a specific compassion meditation to train (*sbyong*) a practitioner to comprehend (*blo*) the ultimate nature of mind and all phenomena. It emphasizes how to see conflicts (and other circumstances that are ordinarily upsetting or depressing) as reasons for happiness in the perspective of dependent origination, e.g., thinking that difficulties faced in day-to-day life are exhausting negative karmic results of one’s own non-virtuous actions in the past; how to transform a self-cherishing attitude into cherishing others, by contemplating the illusory nature of the self, the faults in self-cherishing, and the benefits that flow from cherishing others; the trainings are based primarily on the techniques for equalizing the attunement to self and others and exchange of self and other by taking other’s suffering and giving them self’s happiness (Buswell and Lopez, 2013).

Notably, the exchange of self and other, known as give-and-take (*tonglen, or gtong len*), depends on one’s firm re-appraisal of how one relates to adversities that one experiences, that is, from seeing adversities as unwanted trash to welcoming them as treasury, as it exhausts negative consequences of past non-virtuous deeds, and enhances one’s renunciation of such non-virtues and compassion for others who shared the same experiences, and aspiration for realizing the ultimate nature of mind and all phenomena for the benefits of all, as stated in GBWL (Shantideva, c. 700/1979):

Ch. 6, V.21

Furthermore, suffering has good qualities: through being disheartened with it (SA$\underset{\cdot }{\text{M}}S\bar{\text{A}}$RA is renounced), arrogance is dispelled, compassion arises for those in cyclic existence, evil (non-virtue) is shunned, and joy is found in virtue.

Since adversities can never obstruct the ultimate nature of mind, this is why the transformation of adversities is always possible if one realizes the ultimate nature of mind. On the basis of such transformation, our second hypothesis points to the pith of *lojong* meditation, the equal attunement to self and other’s universal suffering, as stated in GBWL (Shantideva, c. 700/1979):

Ch. 8, V.90

First of all, I should make an effort to meditate upon the equality between self and others: I should protect all beings as I do myself because we are all equal in (wanting) pleasure and (not wanting) pain.

Ch. 8, V.91

Although there are many different parts and aspects such as the hands; As a body that is to be protected they are one. Likewise all the different sentient beings in their pleasure and their pain have a wish to be happy that is the same as mine.

Ch. 8, V.92

The suffering that I experience does not cause any harm to others. But that suffering (is mine) because of my conceiving of (myself as) “I”; Thereby it becomes unbearable.

Ch. 8, V.93

Likewise the misery of others does not befall me. Nevertheless, by conceiving of (others as) “I” their suffering becomes mine; Therefore it too should be hard to bear.

Ch. 8, V.94

Hence I should dispel the misery of others because it is suffering, just like my own, and I should benefit others because they are sentient beings, just like myself.

Ch.8, V.131

If I do not actually exchange my happiness for the sufferings of others, I shall not attain the state of Buddhahood and even in cyclic existence shall have no joy.

#### Prerequisites of Practicing Buddhist Compassion Meditation

On the basis of firm renunciation of ego-preserving bias, a *lojong* practitioner has to first cultivate equality in three levels as prerequisites (Gelek Rimpoche, 2007):

(1) The first level is dwelling on the notion of wishing all beings to be happy and free from suffering.
(2) The second level is the notion of developing equanimity to friends and enemies within one’s self, i.e., inhibiting one’s attraction to friends and repulsion from enemies.
(3) The third level is the notion of *identifying with* friends and enemies *equally* as if their sufferings are one’s own.

Notably, only the third notion, that of identifying with friends and enemies equally, is directly aiming to counter ego-preserving bias. Due to the unwavering equality among self, friends, and foes, this notion of equal identification is therefore not the same as bias-prone *partial* identification, deemed as irrational side effects of empathy (Bloom, 2017), which only further corroborates the necessity of renunciation of ego-preserving bias before practicing *lojong*.

#### Main Practice of Buddhist Compassion Meditation

After the prerequisites, a *lojong* practitioner is ready to cultivate a concentrated meditation on Awakening Mind (BODHICITTA), i.e., the intention to reach the complete, perfect enlightenment, in order to liberate all sentient beings in the universe from suffering. This goal is informed by the recognition of the nature of universal sufferings, as the targets to eliminate, and knowledge of the nature of the mind, as the basis of eventual success. We hereby introduce a technical feature of compassion meditation that is directly relevant to our second hypothesis and explain how it may counter PRAPAÑCA and VIKALPA that cause ego-preserving bias, as follows.

Je Tsongkhapa (1357–1419) taught that a compassion meditation practitioner should focus on the friend, enemy, and those in between, thinking, ‘I’m going to help them’ (Gelek Rimpoche, 2014). Likewise, benevolence (or loving-kindness) meditation is to focus on the friend, enemy, and those in between, thinking, ‘I’m going to make them happy.’ Typically, in these forms of meditations, the three types of people (friends, enemies, and in-between) are visualized in front of the practitioner, and then compassion or benevolence is generated and distributed to them equally.

We postulate that the visualization of friends, enemies, and anyone in between in one’s “meditation field” invokes the mental images of these people, which are preexisting VIKAPLAS in one’s mind. By spatially arranging these three mental images in the “meditation field,” with the images of friends and enemies put on either side and the neutral persons’ image in the center, the inclination to mentally run toward friend or away from enemy is effectively balanced out. By ceasing to have such mental activities of push-and-pull in this visualization process, the conditioning of emotional obsession toward friends, hatred toward enemies, and indifference toward someone in between are gradually weakened and extinguished. In this way, PRAPAÑCA is temporarily suspended and VIKALPA are weakened, from the coarsest type (Types 6–8) to a subtler type of VIKALPA (Types 4 and 5). Ultimately, PRAPAÑCA and VIKALPA as well as resulting identity-grasping belief and ego-preserving bias that are supported by them, can cease to exist when compassion meditation is practiced over time and skillfully mastered.

We hereby summarize a structure of the main practice of compassion meditation below:

(1) Recognize that all people are entrapped in three types of suffering, self and others alike.
(2) Invoke mental images of friends, enemies, and anyone in-between, and arrange these images appropriately in the field of meditation.
(3) Identify with friends, enemies, and anyone in-between *equally*, e.g., their suffering *is* my suffering, no matter who they are.
(4) Think to help them by attuning to their three types of suffering, noticing the resistance from one’s own identity-grasping belief and ego-preserving bias, and let go of them.
(5) Concentrate on this state of mind and let go of conceptual thoughts that emerge, focusing on the ultimate nature of mind until the end of the meditation session.

### Evidence of Buddhist Compassion Practice Effects

The evidence of *lojong* practice’s effectiveness is living in the lives of the practitioners, as demonstrated by many anecdotes. For example, according to a biography of Ribur Rinpoche, a Tibetan monk who practiced and taught *lojong*, he did not lose his compassion and attunement to those who relentlessly tortured him, nor did he feel any psychological sufferings, during his 1959–1976 stint in a Chinese labor camp (Ribur Rinpoche, 1999).

While an experimental trial demonstrating torture-proof effects of compassion meditation is impossible for ethical reasons, a hint of preliminary evidence of our conjecture has been reported in a series of empirical studies testing the effects of a *lojong*-inspired compassion training, Cognitively-Based Cognitive Training (CBCT^®^), which aims to cultivate equal attunement to self and others, although the practice of self-other exchange (*tonglen*) was excluded in its curriculum and post-conflict well-being was not measured as outcome variables in these studies (Pace et al., 2009; Desbordes et al., 2012; Mascaro et al., 2013; Lang et al., 2019; Ash et al., 2020).

## Incorporating Buddhist Concepts in a Bayesian Active Inference Framework

Recent advances in theoretical neurobiology suggest that Bayesian active inference is a universal principle in biological evolution (Campbell, 2016), genotypic phylogeny (Price, 1970), and phenotypic ontogeny (Friston, 2013). According to Free Energy Principle (FEP) (Friston, 2013; Ramstead et al., 2017), an organism is a self-organizing adaptive system, which develops ergodic (self-repeating) models (1) by sensing the features that the environments require the organism to predict and (2) by acting on what the organism expects the environments to be. In so doing, biological organisms capture free energy and thus defy the second law of thermodynamics that requires everything to naturally dissipate into a higher level of randomness. The FEP framework is described in more details in Box 1.

**Free energy principle in biological self-organizing systems.**

By interacting with different entities in the environments, biological systems are self-organizing systems that defy the second law of thermodynamics, which requires that everything naturally dissipates into a higher level of randomness. Thus, biological organisms are inter-dependently self-organizing systems. Karl Friston described Free Energy Principle, as a heuristic model of Bayesian active inference system, capable of drawing random fluctuation and outcomes bounded in a sample space (Friston, 2013). An organism can have many layers of Bayesian Networks. In a Bayesian Network, the relations between nodes can be designated as parent (cause), child (effect), or peer (interaction). If *x* causes *y* to change, then *x* is a parent of *y*, and *y* is a child of *x*; if *x* and y mutually depend on each other, then *x* is a peer of *y*. A Markov Blanket of a node, *x*, refers to a set of variables including *x*’s parents, *x*’s children, and *x*’s children’s other parents. When all the knowledge in *x*’s Markov Blanket is known, then *x* is known.

**FIGURE 1:**
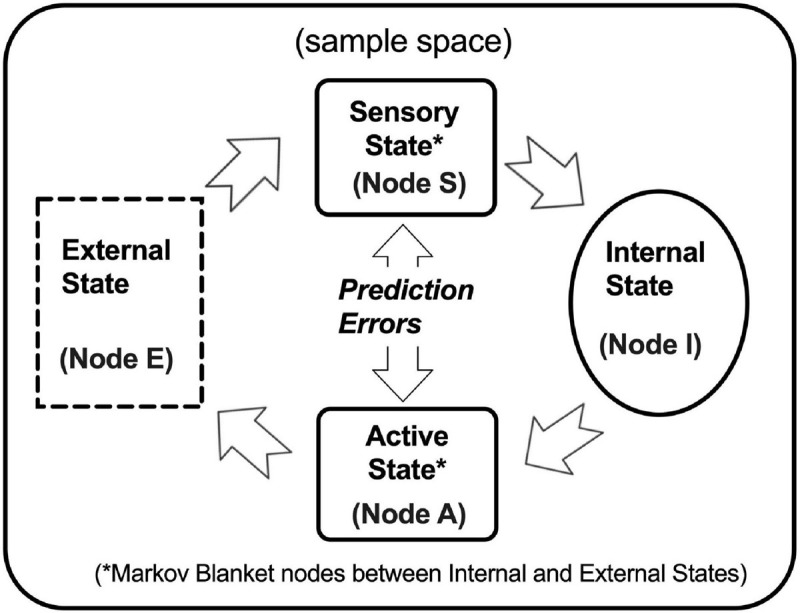
Bayesian active inference framework. Biological organisms are Bayesian Engines, equipped with nodes of Sensory State and Active State (Node S and A, solid round-corner rectangles) and of Internal State (Node I, solid oval), to observe events from the node of External State (Node E, dashed rectangle) in a sample space (bounded by the outer boundary). The causal relations among these nodes follow Free-Energy Principle, described in Box 1.

Free Energy Principle can be heuristically described as follow. As depicted in Figure 1, there are four nodes in a basic Bayesian Network, namely Internal, External, Sensory, and Active States. Internal State is a parent of Active State and a child of Sensory State; External State is a parent of Sensory State and a child of Active State; In addition, Sensory State and Active State are peers, and they are the Markov Blanket of Internal and External States, which means Internal State does not directly observe External State, but it can predict External State through the Markov Blanket. A biological organism is a Bayesian Engine using its Internal State, Sensory State, and Active State to perform Bayesian active inferences of its External State.

(1) External States are the source of observable events that cause sensations and depend on action;
(2) Internal State serves as working models that cause actions and depend on sensations;
(3) Sensory State constitutes a probabilistic mapping from action to external states; and
(4) Active State depends on sensations and working models.

The Bayesian Engine optimizes the inferences actively by updating the prior probability in Internal State to minimize (variational) free energy, the upper bound of prediction error computed as the difference between sensation and actions.

### Karl Friston’s Bayesian Network Model: Free Energy Principle

Although an adaptive system must possess many layers of Bayesian Networks, Friston described a heuristic model with only four nodes, namely Active State (Node A), Sensory State (Node S), Internal State (Node I), and External State (Node E) (Friston, 2013). Every biological organism is a Bayesian Engine, which consists of Nodes I, S, and A, to perform Bayesian active inferences of Node E from the environments. The Bayesian Engine optimizes the inferences actively by computing the prediction error, i.e., the difference between Node S and Node A, and then updating the prior probability in Node I to minimize (variational) free energy, the upper bound of prediction error. See Figure 1.

Thus, a person as a Bayesian Engine can initiate an Internal State as a seed of prior knowledge (prior probabilities) about its environments, and the seed can be transformed into mature “working models” to predict the environments (External State) by utilizing the engine’s sensory receptors (Sensory State) and active effectors (Active State). FEP points to the possibility that the development of a working model—from an initial “seed” of prior probability to its eventual maturity—depends on ongoing optimization of Bayesian active inference, prompted by prediction errors of prior models, but the “life span” of a working model will depend on its capacity in capturing (variational) free energy caused by prediction errors.

### Comparison of Bayesian Network and Five Aggregates

In comparison to Buddhist concepts, we postulate that the *impersonal* Bayesian network may instantiate a network of five aggregates (SKANDHAS). In Buddhist concepts, consciousness (VIJÑ$\bar{\text{A}}$NA) occurs by the conditioning of an internal sense base (INDRIYA) and an external sense object ($\bar{\text{A}}$YATANA). In the Bayesian network, Node E is equivalent to the external sense object $\bar{\text{A}}$YATANA, while Bayesian Engine (Nodes S, I, A) is equivalent to the internal sense base INDRIYA. When the Node E makes a contact (SPARŚA) with the Bayesian Engine, it leads to the feeling (VEDAN$\bar{\text{A}}$), which is equivalent to Node S, of that contact as pleasant, unpleasant, or neutral. Then, Node S leads to Node I (SA$\underset{\cdot }{\text{M}}$JÑ$\bar{\text{A}}$) when prior models are used to make predictions of the events; the predictions cause Node A to act to interact with the events, so Node A is a conditioning factor or volitional action (SA$\underset{\cdot }{\text{M}}SK\bar{\text{A}}$RA). All these nodes are embodied and supported by materiality or form (R$\bar{\text{U}}$PA). In this process, consciousness emerges when the top-down model (Node I) interacts with the bottom-up data (encoded in Nodes S and A) (He and Raichle, 2009; Bachmann and Hudetz, 2014).

Thus, it is relatively straightforward to create potential mappings between Buddhist concepts and the Bayesian framework. Because of this, we are encouraged to use the Bayesian framework to explicate Buddhist concepts in the process related to conflicts and compassion meditation.

### Stress, Resilience, and Free Energy in Bayesian Networks

Since free energy increases when prediction errors increase, and prediction errors increase when one’s expected outcomes conflict with others’ expected outcomes, conflicts tend to increase free energy temporarily until the Bayesian Engine learns to reduce prediction errors by addressing the conflicts. Accordingly, it has been postulated that stress ensues when an organism’s existing working models fail to minimize free energy under challenging circumstances, i.e., uncertainty that threatens one’s prior models (Peters et al., 2017). In other words, as a Bayesian Engine, an individual’s efficacy in re-capturing (and thus reducing) the free energy induced by conflicts is directly related to the individual’s stress resilience. The notion that the exposure and subsequent reduction of conflict promote stress resilience is highly consistent with the stress inoculation hypothesis, supporting that brief intermittent stress exposure in early life can induce relatively low stress reactivity to stressors in adulthood in humans and non-human primates (Parker et al., 2006).

### The Embodiment of Bayesian Engine

According to FEP, the development of an individual organism (ontogeny) is a dynamic process of individual-environment transactions that gradually *embody* the functional nodes of the Bayesian Engine. The embodiment of Bayesian Engine is probably corresponding to the notion of clinging to physical existence depending on conceptual imputation, i.e., dependent designation (PRAJÑAPTI UP$\bar{\text{A}}D\bar{\text{A}}$NA). Once embodied, the nodes serve as conditioned circumstances that interact with the environments to register features of an incoming event (via Nodes S and A), as a primary transaction, and then to infer how the Bayesian Engine should relate to the event (via Node I), as a secondary transaction. The embodiment of these nodes makes these primary and secondary transactions *automatic*, such that the agent subjectively experiences phenomena without any efforts. In other words, the agent automatically takes an observer perspective when perceiving events that seem to occur separately from one’s viewpoint, hence the split between the observer and the observed (i.e., the emergence of the duality in the mind).

The embodiment of Bayesian Engine does not entitle any intrinsic independent existence of an individual agent. When multiple agents interact in a multi-agent system, each agent is not only a subject of his or her own experience but also an object for other agents. In other words, all phenomena that can be observed must be originated from the interactions among multiple Bayesian Engines. Because none of the nodes in any Bayesian Engines can be a constant (i.e., non-variable), otherwise it will be drop out of mathematical operations that give rise to any phenomena, there cannot be any permanent observer (self) or observed objects (others). Since all nodes are inter-dependent in any Bayesian networks, there is no inherently individual entity as a Bayesian Engine. For a discussion on the limitation of Darwinian self-preservation interpretation in prosocial behaviors, see Ludwig and Welch (2019). We postulate that the self-preservation interpretation should be replaced by the notion of attunement to the environments, including one’s counterparts in conflicts.

### VIKALPA Mediated the Reification of Self and Other’s Divided Identities

**FIGURE 2:**
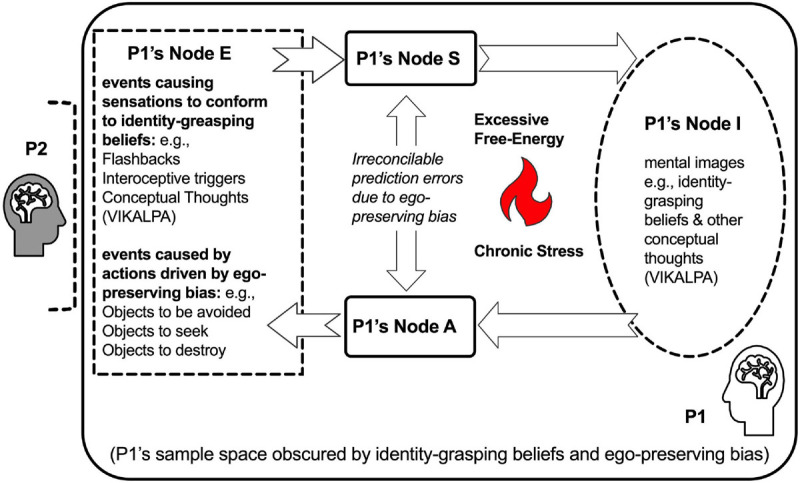
Bayesian Engine in a state that identity-grasping beliefs and ego-preserving bias obscure its sampling space. When identity-grasping beliefs and other conceptual thoughts (VIKALPA) “hijack” a person P1’s Node I, these conceptual thoughts explain away prediction errors without attuning to another person P2. The reactions to the events from Node E pre-occupy P1’s Nodes S, A, and I at the expense of actual attunement to P2’s Bayesian Engine, manifesting as an ego-preserving bias. Outwardly, the identity-grasping beliefs in P1’s Node I drive P1’s Node A (escape, aggression, or acquisition) to avoid fear-provoking objects and/or seek distracting or addictive objects with negative reinforcement; Inwardly, the sampling of the events is biased toward covert cues, e.g., trauma-related flashbacks, interoceptive triggers, and conceptual thoughts (VIKALPA) that cause fear, anger, or obsession in P1’s Node S to re-confirm the conceptual thoughts (VIKALPA) in P1’s Node I. In such an obscured state, P1 fails to infer and “see” the other person in the relationship, denoted by the dashed bracket in front of P2. The obscuration of P1’s Bayesian Engine is denoted by the other person P2 who is excluded from P1’s sample space. Due to the conflicts that are not constructively reduced by the obscured Bayesian Engine, excessive free energy ensues and results in chronic stress.

By the age of two a child normally develops object permanence, i.e., a phenomenon wherein a child starts to seek an object when it is not sighted surprisingly (Shaffer and Kipp, 2013). The development of object permanence suggests the emergence of mental image that mediates the child’s object seeking, implicating an operation of conceptual thoughts (VIKALPA). Because phenomena appear to the observer so automatically and reliably, the observer may likely neglect the mental images mediating the object permanence of self and others, such that identities of self (the observer) and others (the observed) are reified as truly existing, i.e., identity-grasping beliefs. In so doing, the observer may over-reduce others (the observed) to mental objects, neglecting that other people are also living Bayesian Engines who can actually feel (equipped with Node S), do (Node A), and think (Node I). See Figure 2.

We wish to underscore that identity-grasping beliefs are not “innately” built-in features of any Bayesian Engines. As demonstrated by the success of artificial intelligence programs, these artificial Bayesian Engines are *impersonal* and free of any identity-grasping, as they never refuse to update any prior models (Silver et al., 2017a,b). Thus, it is clear that identity-grasping beliefs are adventitious not only to the nature of mind, but also to the operations of Bayesian Engines.

## A Brain Model for the Social Cognition, Embodied Identity-Grasping Beliefs, and Compassion Meditation Effects

The human neuroimaging literature suggests that mental factors (not the ultimate nature of mind *per se*) can be related to the brain from the perspective of *neurophenomenology* (Varela, 1996). Here we propose a putative brain model to contemplate how five-aggregates-related Bayesian Engine is supported by the brain, and how the brain responds to conflicts in a bifurcating way, either disturbed by identity-grasping beliefs or not. We postulate that *lojong-like* compassion meditations would shun the influences of identity-grasping beliefs (hence ego-preserving bias) in the brain. Notably, this postulation does not necessarily support the notion that the brain is a physical cause of mind. Rather, the putative brain model is like a model of two modes of brain in response to conflicts. Using the clear lamp shade metaphor, the model of brain is not a model of clear lamp shade (the nature of mind), but a model of two light bulbs (two modes of mental factors), in which one light bulb would color the mind with the ego-preserving bias and the other would not.

### A Brain Model in Bayesian Framework

**FIGURE 3:**
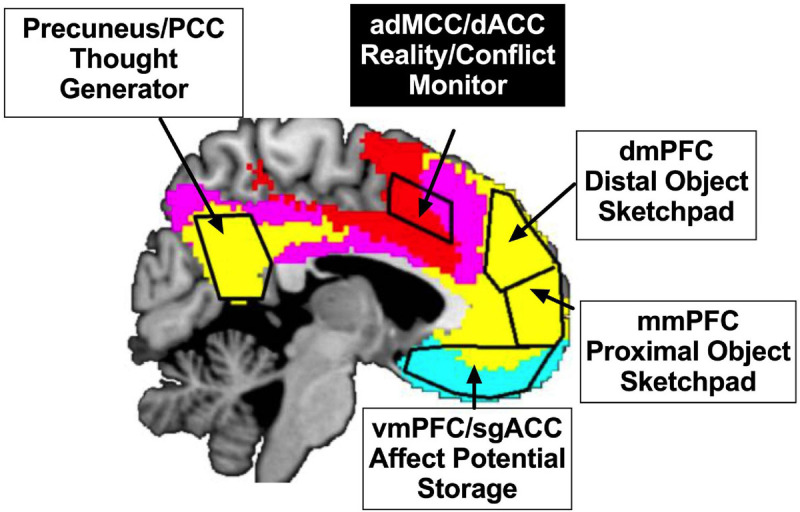
Affect-object generative inference and regulation (AGIR) model, adapted from Ho and Nakamura (2017). There are two systems in the model. In the first system, an affect-object inference generation system encompasses four components: a thought generator in precuneus/posterior cingulate cortex (PCC), an affect potential storage in subgenual anterior cingulate cortex (sgACC) for negative affect and ventromedial prefrontal cortex (vmPFC) for positive affect, a proximal object sketchpad in middle medial prefrontal cortex (mmPFC), and a distal object sketchpad in dorsomedial prefrontal cortex (dmPFC). When “vehicles” of conceptual thoughts are running in this system, thoughts of affect-objects can be represented as mental objects (e.g., self = proximal objects charged with affective potentials; other = distal objects charged with affective potentials). In the second system, a cognitive control system encompasses a component of reality/conflict monitoring in anterior dorsal middle cingulate cortex (adMCC)/dorsal anterior cingulate cortex (dACC) and other cortical components not depicted in the figure. The main function of reality/conflict monitoring is to pause an ongoing affect-object generating thought and observe all modalities of information involved in a potential conflict, allowing an unrealistic expectation generated in the first system to be paused and updated. In terms of cortical networks that are self-organized in intrinsic resting-state functional connectivity in Yeo et al. (2011), the first system primarily involves Default Mode Network (yellow) and Cortical Limbic Network (cyan), and the second system primarily involves Ventral Attention Network (red) and Frontal-Parietal Network (violet). The colors of the components’ text boxes are reversed between the two systems, indicating an anti-correlative relationship between them. The demarcation of the brain areas is approximate.

The Bayesian active inference framework has been applied to several domains, e.g., self-reference (Carhart-Harris and Friston, 2010), interoception (Barrett and Simmons, 2015), visuo-motor mirror-neuron system (Kilner et al., 2007), and post-conflict well-being (Connolly, 2018). We reported a meta-analysis to explain a post-adversity bifurcation in the brain responses, highlighting a brain network, namely the Affect-Object Generating Network, through which conceptual thoughts can be generated by linking the representations of self (as a proximal object) and others (as a distal object) to affective potentials via thinking processes propelled by a thought generator, depicted in Figure 3 (Ho and Nakamura, 2017).

**FIGURE 4:**
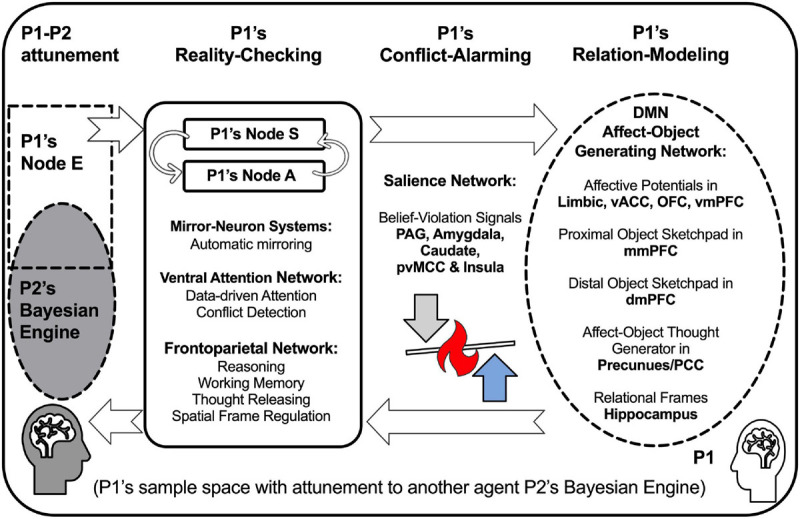
Bayesian Engines in a state of attuning to others, such that all agents (e.g., P1 and P2) in the system are present in the sampling space of their Bayesian Engines. Here, we focus on P1’s Bayesian Engine as an example, even though P2’s Bayesian Engine is also present in the same sampling space (denoted by the inclusion of P2 in P1’s sample space). The Bayesian Engine consists of three functional components, namely Relation-Modeling, Reality-Checking, and Conflict-Alarming. Relation-Modeling is a component, consisting of Node I, wherein conceptual thoughts involving self and others are generated in the Affect-Object Generating Network (part of Default-Mode Network, DMN) in a relational frame generated by hippocampus and parahippocampal cortex. Reality-Checking is a component, consisting of Nodes S and A, subserved by multiple brain networks for data detection, reality monitoring, and attention regulation, including the Mirror-Neuron System, Ventral Attention Network, and Frontoparietal Network. Conflict-Alarming is a component that can be triggered by excessive free-energy due to conflicts that violate one’s rigid beliefs, so conflicts can activate the brain regions in the Salience Network, including periaqueductal grey (PAG), amygdala, caudate nucleus, posterior ventral middle cingulate cortex (pvMCC), and anterior insula. We postulate that when a conflict between P1 and P2 occurs, P1’s Conflict-Alarming is triggered, then P1’s subsequent course of actions can bifurcate, pivotally depending on how P1’s Salience Network regulates other brain networks underlying Relation-Modeling and Reality-Checking, denoted by the up/down arrows near the flame: Post-conflict resilience will ensue if the activations of Salience Network consistently down-regulate pre-existing Relation-Modeling and up-regulate Reality-Checking, such that P1 can attune to P2 adaptively, which will ultimately reduce conflicts in the long run; conversely, identity-grasping beliefs and ego-preserving bias will be exacerbated if the activations of Salience Network consistently up-regulate pre-existing Relation-Modeling and down-regulate Reality-Checking, which will generate even more conflicts in the long run.

We hereby propose a putative brain model of social cognition in the Bayesian framework, as depicted in Figure 4. To simplify the description, we will first parse the brain-based Bayesian Engine into three functional components, namely Relation-Modeling, Reality-Checking, and Conflict-Alarming. As seen below, these three functional components are large-scale brain networks that support complex mental processes involved in social cognition in general and cognitive processing of potential conflicts in particular.

(1) Relation-Modeling refers to the development and selection of Relational Models in the Bayesian Engine’s Node I (Internal State). It is primarily subserved by the hippocampus and the Default-Mode Network (DMN) that consists of the precuneus, ventromedial prefrontal cortex (vmPFC), orbitofrontal cortex (OFC), (anterior) middle medial prefrontal cortex (mmPFC), dorsomedial prefrontal cortex (dmPFC), perigenual ventral anterior cingulate cortex (vACC), temporal poles, and parietal lobes. We will discuss the functional significance of these regions in Section “Relation-Modeling: Generating Affect-Objects in Conceptual Thoughts (VIKALPA).”
(2) Reality-Checking refers to the detection and regulation of data-driven, primary transactions in the Bayesian Engine. It is primarily subserved by the following frontoparietal areas: (a) the Mirror-Neuron System that automatically detects the intention of others’ actions in premotor and parietal areas (Iacoboni et al., 2005), and this Mirror-Neuron System overlaps with the DMN in the parietal lobe; (b) the Ventral Attention Network (VAN) that monitors features of episodic events (reality monitoring), prediction errors, and response conflicts; (c) the Frontoparietal Network (FPN) that performs higher order cognitive control including working memory that maintains or updates the contents as mental objects (Cohen et al., 1997), and FPN is involved in releasing a set of rules or thoughts according feedback (Buchsbaum et al., 2005) and in spatial frame processing to represent objects according to ego-centric or allocentric (other-centered) references in space. This FPN overlaps with the DMN in the hippocampus, parahippocampal cortex, and retrosplenial cortex (Ekstrom et al., 2014). We will discuss some key functions of this component in more details in Section “Reality-Checking: The Gateway to Attuning to Self and Others.”
(3) Conflict-Alarming refers to the intense responses when an individual perceives threats to his or her prior model, as a *special case* of severe prediction error signals in the Bayesian Engine. The Conflict-Alarming component is primarily subserved by the Salience Network (SN), which includes dorsal anterior cingulate cortex (dACC), posterior ventral middle cingulate cortex (pvMCC), bilateral anterior insula/orbital frontal insula, subcortical regions such as periaqueductal gray (PAG), hypothalamus, thalamus, midbrain, striatum (including caudate), and extended amygdala (Seeley et al., 2007). While SN and VAN overlap in several cortical areas, as they are both involved in the processing of prediction error signals, the substrate of Conflict-Alarming component refers to the part of SN that is preferentially activated in pain, i.e., intense emotional responses related to belief-violating or survival-threatening prediction errors (Lieberman and Eisenberger, 2015). According to the public online database providing automatic term-based meta-analysis of neuroimaging studies (Yarkoni et al., 2011), brain regions that are preferentially associated with the term “pain” include the SN mentioned above, and additionally some regions overlapping with the anterior midline regions in DMN (mmPFC, vmPFC, and vACC) and the lateral regions of FPN/VAN (right prefrontal and bilateral parietal lobes). The SN network largely overlaps with the parental brain (Ho et al., 2014; Swain and Ho, 2017) and the SN (amygdala and frontoinsular) responses to infant cry was associated with mother–child intersubjectivity in free play (Hipwell et al., 2015) pointing to the roles of parenting in the development of compassion (Swain et al., 2013). We will discuss some key functions of this component in more details in Section “Conflict-Alarming: The Salience Network at the Pivotal Point.”

In what follows, we provide more detailed functional characterizations of the three components in the Bayesian Engine in the context of conceptual thoughts (VIKALPA), attunement, and post-conflict bifurcation.

#### Relation-Modeling: Generating Affect-Objects in Conceptual Thoughts (VIKALPA)

Some evidence for the designation of these regions’ functions is described below. The functional distinction of the affective potential, proximal object sketchpad, and distal object sketchpad is highly consistent with a body of literature showing how the respective brain regions (the vmPFC, mmPFC, and dmPFC) are functionally connected to other brain regions distinctively (Li et al., 2014). The relative distinction between the proximal and distal object sketchpads is that the former can represent one’s self and the latter can represent remote objects (animate or inanimate) during mentalization in the mmPFC and dmPFC, respectively. A meta-analysis suggested that self- and other-oriented mentalizations are mapped onto the medial prefrontal cortex with a spatial gradient in the direction from the mmPFC (self-oriented) to the dmPFC (other-oriented) (Denny et al., 2012). When individuals had more gripping experiences during the retrieval of negative autobiographical memories, the mmPFC activation was increased accordingly (Kross et al., 2009). Anatomically, as compared to the dmPFC, the mmPFC (also known as BA10p) is more intimately connected to interoception-processing areas such as the insular, vACC, vmPFC, and OFC (Petrides and Pandya, 2007). The distinction is further supported by a study using machine learning algorithm in neuroimaging to validate their roles across different tasks (Skerry and Saxe, 2014), which documented that both mmPFC and dmPFC can represent cross-modal, abstract values, rather than stimulus-driven values (consistent with the notion that both mmPFC and dmPFC are “sketchpads” that can represent abstract information) and the mmPFC, not dmPFC, can also represent subjectively experienced values (consistent with the distinction of proximal versus distal representation between the two regions); furthermore, the vmPFC can represent subjectively experienced values, rather than abstract values (consistent with its role as affective potential).

In addition to the medial prefrontal cortex, the precuneus/PCC is considered to play a role in thought generation, suggested by the converging evidence that the precuneus/PCC region is associated with the terms of “thinking” or “thoughts” (Ho and Nakamura, 2017). Indeed, in a neurofeedback study based on the activity level of precuneus/PCC, this region became more active when the participants exerted more efforts in thinking while they performed a self-regulation task and conversely, the region became rested when they achieved a state of mental quiescence (Garrison et al., 2013).

Thus, the Relation-Modeling component is supported by the combination of the Affect-Object Thought Generation—subserved by the amygdala, precuneus, vACC, vmPFC, OFC, mmPFC, and dmPFC—and the Relational Framing—subserved by the hippocampus, parahippocampal cortex, retrosplenial cortex, and parietal lobe. The relation frames provide a temporal-spatial context in which an individual is virtually situated in, and in this context the precuneus/PCC can produce temporal affect-objects by generating thoughts that “affectively color” the representations of self or other in the sketchpads of proximal object (mmPFC) or distal objects (dmPFC), respectively, by channeling the affective potentials (mediated in the amygdala, vACC, OFC, and vmPFC) to these sketchpads, leading to the formation of affect-objects. Under the influences of identity-grasping beliefs, the conceptual thoughts (VIKALPA) regarding the identities of self and others can influence the precuneus/PCC that may serve as the generator of thought (VITARKA).

#### Reality-Checking: The Gateway to Attuning to Self and Others

The Reality-Checking component should involve the VAN and FPN: the former can monitor background information and inhibit ongoing responses when surprises (prediction errors) happen, and the latter can make information explicitly available in one’s awareness. For examples, FPN forms a coherent coupling with another network containing the information (Cole et al., 2013). When individuals become subjectively aware of their spontaneous thoughts generated in Affect-Object Generating Network, they will have co-activation of FPN and DMN (Fox et al., 2015); likewise, the FPN’s connectivity with DMN was strengthened when the participants retrieved autobiographical episodic memories from the past or imagining a future scene (both tasks activating the DMN), but not when they were performing visual-spatial tasks unrelated to the DMN (Schacter et al., 2012).

Here, we focus on the adMCC, as it plays an integral role in functions related to both VAN and FPN, e.g., conflict monitoring, working memory, and inhibition (de la Vega et al., 2016). The adMCC region has been specifically identified as a region in which the gray matter structural property is directly associated with reality monitoring performance in a memory task, which required monitoring memory retrieval and inhibiting distractors (Buda et al., 2011). The individual differences in the morphology of adMCC are related to the capacity for the regulation of negative emotions (Shackman et al., 2011).

Notably, the roles of the reality-monitoring adMCC in FPN/VAN and the distal object dmPFC in DMN are dissociable in social cognition. In an empirical study (Sommer et al., 2007), the dmPFC in DMN was more activated when individuals predicted what others might do on the basis of a belief that was identical with the self’s perspective (undifferentiated belief), as compared to what they might do on the basis of a differentiated (individuated) belief, when the other’s perspective would be opposite to the self’s; conversely, the regions in FPN/VAN, including the dACC/adMCC, right prefrontal cortex, right temporal parietal junction, and right dorsal precuneus were more activated in differentiated belief than in undifferentiated belief conditions. This study provides supportive evidence that the dmPFC represents another person’s attributes as a distal object in an ego-centric frame (which is VIKALPA-dependent), while the dACC/adMCC is needed when one represents others in an allocentric frame with differentiated perspectives. Interestingly, always seeing another person in an ego-centric frame is considered a symptom of under-developed self with inadequate understanding of others (Kohut, 1982; Summers, 2014). Thus, the Reality-Checking component of the Bayesian Engine, mediated by the FPN/VAN, can be considered a gateway to facilitate engagement with others effectively, as it enables the person to see self and other’s subjectivity flexibly.

#### Conflict-Alarming: The Salience Network at the Pivotal Point

We postulate that excessive free energy should trigger the SN underlying the Conflict-Alarming component, thus the SN should play a pivotal role in regulating the dynamic coherence between the DMN and FPN/VAN underlying Relation-Modeling and Reality-Checking components, respectively.

In accordance with this notion, it has been found that the SN can initiate the up- or down-regulation of DMN and FPN/VAN (Goulden et al., 2014). In addition, the striatum, as part of the Conflict-Alarming component, also plays a pivotal role in regulating DMN and FPN. In a healthy state, violation of expectations (a form of prediction error) can activate the caudate (O’Doherty et al., 2004) and the caudate was associated with urge-to-react (Jackson et al., 2011). The urge to smoke increased response in the vACC and vmPFC along with increased craving for nicotine in smokers (Wilson and Sayette, 2015). Patients with Obsessive Compulsive Disorder (who have excessive urge-like thoughts or behaviors) showed excessive coupling between the caudate and the vACC, vmPFC, and dmPFC (Harrison et al., 2009). When facing a fear-evoking situation, a stronger coupling between the caudate and vACC (part of DMN) resulted in a greater urge to react with unnecessary but prepotent responses, while a stronger coupling between the caudate and right prefrontal cortex (part of FPN/VAN) resulted in the inhibition of the urge to act out the unnecessary reactions (Gillan et al., 2015). Thus, the caudate can up- or down-regulate FPN and DMN motivationally. When healthy individuals were challenged by acute stressors in the laboratory, their adaptive coping responses increased when the SN increased its functional connectivity with the FPN, but their negative affects and ruminative thoughts increased when the SN increased its functional connectivity with the DMN (van Oort et al., 2017).

The SN may fail to down-regulate the DMN when one’s mental states are associated with exacerbated ego-preserving bias. For example, after trauma-related episodes were triggered, the functional connectivity among the amygdala (part of SN) and vACC and vmPFC (part of DMN) was hyperactive in patients with PTSD (Dunkley et al., 2014). The integrity of SN, measured as the white matter tractography between the right anterior insula and dACC, was associated with the capacity for response inhibition, which was achieved by deactivating DMN when receiving a signal to stop a response (Bonnelle et al., 2012). Indeed, the dACC and anterior insula in the SN are of great clinical significance because structural and functional deficits of these regions are commonly identified in multiple major psychiatric disorders including major depression, anxiety, obsessive-compulsive (OCD), substance use, bipolar, and schizophrenia disorders (Goodkind et al., 2015).

### The Brain Model Underlying Post-conflict Bifurcation

According to our model, prior models supported by conceptual thoughts of self and others (VIKALPA) are subserved by DMN in the Relational Modeling component. Reality is registered and prediction errors are detected by FPN/VAN in the Reality-Checking component. When interpersonal conflicts contradict the prior model, SN in the Conflict-Alarming component is triggered. This Bayesian model is consistent with a recent meta-analysis on brain responses to viewing faces of same or different races (Bagnis et al., 2020). In this meta-analysis, own- and other-race visual categorizations led to distinct activation patterns in the brain: Categorization of own-race mainly activated DMN and categorization of other-race mainly activated visuo-attentive processing and amygdala (part of SN) (Bagnis et al., 2020). We interpret these meta-analysis results as consistent with our model, because the same-race stimuli are relatively familiar and similar to the prior models stored in DMN, so these stimuli can mainly activate DMN; and the other-race stimuli are relatively unfamiliar and dissimilar to the prior models, so they may carry more prediction errors (relative to the same-race stimuli) to activate the SN and other reality-checking regions.

We hereby describe how this brain model can explain the post-conflict bifurcation between maintaining the attunement and exacerbating ego-preserving bias. As described in the section above, when excessive free energy resulting from conflicts activates the Conflict-Alarming component, the underlying SN plays a key role in the regulation of what behavior will follow afterward. When the SN down-regulates the DMN and up-regulates FPN/VAN, the person can inhibit the previous Relational Models and potentially engage more productively with the ongoing demands from the environments. When the SN fails to down-regulate DMN, the Bayesian Engine would not be able to use the excessive free energy to inhibit ongoing prepotent Relational Models subserved by DMN, and then the person is susceptible to urges to act out automatic habitual behaviors that are entangled with prior models instantiated in the Bayesian Engine. In other words, in the latter case, the individual will exacerbate the ego-preserving bias, which manifests as the inability to update one’s prior models or beliefs in the presence of salient prediction errors during the conflicts with others.

The notion that SN’s post-conflict up-regulation of DMN can indicate a potential exacerbation of ego-preserving bias is consistent with a recent neuroimaging study on implicit racial bias in samples of Black and White Americans (Wang et al., 2019). As shown in this study, both Black and White participants rated other people’s social encounters less favorably when the social encounters were cross-race, involving one Black and one White person, as compared to those same-race encounters involving two persons of the same race; both Black and White participants showed reduced reward activities, indicating negative values as prediction errors in rewards, and enhanced activities in the temporal-parietal junction in the DMN when viewing the cross-race encounters as compared to same-race ones (Wang et al., 2019).

In addition, the ego-preserving bias may be exacerbated when certain overlapping regions between the Relation-Modeling and Reality-Checking components are functionally impaired. These regions include the parietal lobe, hippocampus and adjacent hippocampal cortex, and retrosplenial cortex, which are all closely related to spatial frame processing and regulation. These overlapping regions are known to mediate the regulation of egocentric-allocentric frames in a mental space, which is key to interpersonal understanding and resilience to adversity (Frith and de Vignemont, 2005; Gilbertson et al., 2007; Langston and Wood, 2010; Ekstrom et al., 2014). As evidenced in animal models (Hammels et al., 2015), hippocampus and related regions are crucial regions mediating the bifurcation of post-trauma stress disorders and resilience. The hippocampus is crucial for pattern differentiation and contextual frame completion. The neurogenesis in hippocampus is crucial for the resilience in chronic stress and mood regulation through enhancing cognitive flexibility (Anacker and Hen, 2017), and social experiences can modulate the hippocampal neurogenesis (Dranovsky et al., 2011). Conversely, the impairment of hippocampus will compromise contextual frame processing, e.g., allocentric spatial framing, (Langston and Wood, 2010). In human twin studies, the weaker capacity of hippocampus-dependent allocentric framing is a dispositional risk factor for developing posttraumatic stress disorders (Gilbertson et al., 2007).

### The Relations Between Compassion Meditations and the Brain Model

Here we postulate some predictions of compassion meditation effects on the brain based on our Bayesian brain model, discussed above. First, we predict that compassion should inhibit conceptual thoughts (VIKALPA) that are generated in the affect-object generating network, which is largely overlapping with DMN, as part of the Relation-Modeling component. This is consistent with a neurofeedback study showing that the more one can rest the precuneus/PCC, which may be a generator of active conceptual thoughts in the DMN, the calmer and peaceful the participants can be in the study (Garrison et al., 2013).

Second, we predict that the compassion meditation can enhance the attunement to others through enhancing the reality monitoring capacity or volume in the adMCC (Buda et al., 2011), as the volumes of adMCC are related to the capacity for the regulation of negative emotions (Shackman et al., 2011). This is consistent with the meta-analysis of meditation, showing that the activity in the adMCC is enhanced by both open monitoring meditation that improves awareness of spontaneous mental phenomena and single-pointed meditation that improves attentional focus and stability (Fox et al., 2016).

Third, we predict that the compassion meditation can enhance the capacity in the FPN/VAN as part of the Reality-Checking component through enhancing the attunement to others’ feelings. This is consistent with several studies showing that long-term meditation practitioners have increased cortical thickness in the frontal-insular cortices, and greater responses in these regions when performing loving-kindness meditation, as compared to meditation-naive controls (Engen et al., 2018). Interestingly, enhancing the attunement to others’ feelings may be different from feeling painful when observing other people in pain. In a neuroimaging study, the long-term meditation practitioners, who practiced mindfulness meditation just prior to observing others’ social pain, showed reduced left anterior insula activation during the observation of others’ pain, as compared to meditation-naive controls, and the strength of the anterior insula activation following the mindfulness meditation was negatively associated with levels of trait compassion in these long-term meditation practitioners (Laneri et al., 2017). While these results may suggest that concentration meditation could reduce potential vicarious distress due to the observation of others’ suffering, an alternative explanation may be that the practitioners have reduced their alarming signals, as the anterior insula is a key region in the SN, as part of the Conflict-Alarming component, because they have reduced ego-preserving bias when observing others’ suffering.

Lastly, as described above, the hippocampus and adjacent areas play a critical role in the bifurcation of post-trauma mental disorders and resilience (Hammels et al., 2015), possibly by promoting the neurogenesis in hippocampus that can enhance cognitive flexibility (Anacker and Hen, 2017). Furthermore, a human twin study suggested that the weaker capacity of hippocampus-dependent allocentric framing is a dispositional risk factor for developing posttraumatic stress disorders (Gilbertson et al., 2007). Thus, we predict that compassion meditation should help promote the capacity in the hippocampus and adjacent areas by its spatial arrangement of the visualization of the mental images of friends, enemies, and anyone in between. Specifically, we predict that compassion meditation can promote neurogenesis in the hippocampus after conflicts or other anxiety-provoking situations. This prediction is consistent with a randomized controlled longitudinal study: (1) after mindfulness meditation training, the volume increases in the left subiculum (potentially due to neurogenesis in the hippocampus) were associated with decreased coupling between the left hippocampus and two clusters in lateral occipital cortex, and (2) the larger decreases in the hippocampus-occipital functional coupling were associated with the larger decreases in anxiety levels after the meditation training (Sevinc et al., 2020).

### The Neuroscience of Compassion Meditations

Neuroimaging research in the neural correlates of compassion meditation is still in its infancy. While more studies are needed to test our predictions described above, results from some studies on neuroscience of compassion meditation or similar training are described here. In a meta-analysis of functional neuroimaging research in compassion based on 16 fMRI studies reviewed, the shared brain regions that are consistently related to compassion were identified, including the PAG, bilateral anterior insula, and putamen, which are part of the SN, the dorsal ACC/adMCC, which is part of the FPN/VAN, and the inferior frontal gyrus and subgenual ACC, which are part of DMN. This meta-analysis failed to identify regions purportedly common to compassion such as the DLPFC, OFC, and amygdala, possibly due to the inclusion of a small number of studies that used loving-kindness meditation in their meta-analysis (Kim et al., 2020).

Specifically, a 2-week compassion training (focusing on benevolent wishes for family, friends and difficult people) was found to have increased altruistic behaviors, which were associated with altered activation in the inferior parietal cortex and dorsolateral prefrontal cortex (DLPFC), and in DLPFC connectivity with the nucleus accumbens (Weng et al., 2013). In a randomized study investigating effects of 2-week compassion vs. reappraisal training (Weng et al., 2018), neural responses were measured before and after training while participants actively engaged in their assigned training in response to images depicting human suffering or non-suffering; the study found that increases in the amount of time looking at aversive images due to compassion training were associated with decreases in the amygdala responses. The authors of the study suggested that compassion meditation can cultivate an enhanced motivation to look at visual images depicting suffering while attenuating amygdala responses to the aversiveness of stimuli. However, an alternative interpretation may be that the compassion training increased the synchrony between the negative valence of the pictures and the valence-dependent responses in the amygdala, as suggested by a recent study on compassion-promoting parenting intervention effects on amygdala (Ho et al., 2020).

As mentioned earlier, a *lojong*-inspired compassion training showed beneficial effects on stress reactivity and the brain (Pace et al., 2009; Desbordes et al., 2012; Mascaro et al., 2013; Lang et al., 2019; Ash et al., 2020). In a longitudinal study (Singer and Engert, 2019), distinct constructs of attention and interoception (present-moment), socio-emotional processes (compassion and loving-kindness), and meta-cognitive processes (perspective-taking), were evaluated and found to be differentially augmented in the 9-month training program; generally, the results supported differential neural plasticity that promoted differential training effects on social cognition, altruism, and hormonal response to psychosocial stress. Notably, we recently reported how a compassion-promoting parenting intervention in mothers promoted the attunement to their child through the changes in the brain network in support of our predictions, e.g., better maternal attunement to the child was associated with decreased parenting stress and increases in attunement-dependent responses in the amygdala and reality-checking networks; the training also decreased dmPFC responses that may mediate child-specific conceptual thoughts when the mothers responded to the child; the decreases in functional connectivity between PAG and dmPFC were associated with decreases in parenting stress, suggesting that maternal stress was reduced when relatively less defensive signals from PAG were channeled to the mothers’ mental images of the child, which would have “stained” the maternal perception of the child (Ho et al., 2020).

## Conclusion

In this paper, we aimed to expand the science of compassion in multiple aspects: (a) we expanded the scope of the science of compassion by suggesting that the practice of compassion is an intervention to recognize the ultimate nature of mind and promote post-conflict well-being, over and beyond studies of social emotions and prosocial motivations; (b) we expanded the theoretical background of compassion science by importing Buddhist concepts; (c) we expanded the translational framework of compassion science by juxtaposing the concepts in Buddhism, Bayesian active inference, and Neuroscience in a unified framework. We hope that the present paper may serve as an example of interdisciplinary contemplative science investigation that can stimulate and facilitate cross-disciplinary integrations in compassion research.

“Compassion is not religious business; it is human business; it is not luxury; it is essential for our own peace and mental stability; it is essential for human survival.” – HH the 14th Dalai Lama.

The turmoil of COVID-19 pandemic, United States presidential election, and geopolitical struggles happening in year 2020 may have marked a transformative opportunity for the humanity to bifurcate into future actions. Will the humankind traverse a right course of actions into the future? Our theoretical work may support the notion that compassion is a necessity, not a luxury, for the survival of the humanity, as quoted above. We wish to present this work as a small steppingstone toward the humanity’s evolution into a future where conflicts in all levels of the society can be addressed with compassion and kindness by as many people as possible, i.e., doing our best to recognize the ultimate nature of mind and universal suffering caused by ego-preserving bias and rather wishing to attune to all people equally, friends and enemies alike.

## Data Availability Statement

The original contributions generated for this study are included in the article/supplementary material, further inquiries can be directed to the corresponding author.

## Author Contributions

SH is the principal developer of the theoretical framework and hypotheses and writer of the manuscript. He created the figures in the study. YN collaborated with SH in developing and refining the theoretical framework. He also co-wrote the manuscript. JS co-wrote the manuscript and supported the research involved in the manuscript. All authors contributed to the article and approved the submitted version.

## Conflict of Interest

The authors declare that the research was conducted in the absence of any commercial or financial relationships that could be construed as a potential conflict of interest.
